# Editorial: Insights Into Acromegaly Complications

**DOI:** 10.3389/fendo.2022.905145

**Published:** 2022-06-27

**Authors:** Corin Badiu, Przemyslaw Witek

**Affiliations:** ^1^ Department of Endocrinology, Faculty of Medicine, “Carol Davila” University of Medicine and Pharmacy, and National Institute of Endocrinology, Bucharest, Romania; ^2^ Department of Internal Medicine, Endocrinology and Diabetes, Mazovian Brodno Hospital, Warsaw Medical University, Warsaw, Poland

**Keywords:** acromegaly arthropathy, heart failure, diabetes, high blood pressure, sleep apnoea

Acromegaly is a rare endocrine multisystemic disorder, caused in the vast majority of cases by a somatotroph pituitary adenoma. The excess secretion of growth hormone (GH) and consequently, of insulin like growth factor 1 (IGF1) cause many physio-pathological changes in virtually all organs and tissues (osteoarticular system, muscles, brain, heart and blood vessels, respiratory and hematopoietic system, kidneys, liver and pancreas, thyroid, adipose tissue). In addition, several important complications might appear due to adenoma compression on neighboring neural anatomic structures, upon optic pathway or oculomotor nerves from the cavernous sinuses. Compression upon the normal pituitary tissue could lead to associated pituitary insufficiency. Excess of GH and IGF-1 trigger well documented colonic and thyroid neoplastic changes. All these are considered acromegaly complications and deserve a detailed, personalized approach.

Epidemiological data shows that acromegaly is diagnosed with an incidence of 3.8 cases per million, with a prevalence of 6 per 100.000 (1). However, confirmed in most studies, the long evolution until diagnosis explains the high prevalence of acromegaly complications. The highest impact upon survival is related to cardiovascular complications, while the quality of life is impacted mostly due to chronic degenerative osteoarticular changes (2).

Currently, the selective transsphenoidal adenomectomy is the first line treatment, followed by medical therapy using somatostatin receptor ligands (SRLs) (3), dopamine agonists or GH receptor blocker pegvisomant. Radiotherapy is used in selected cases, especially in resistant, invasive adenomas. Presurgical treatment with SRLs could be used in selected cases, for a better hormonal and/or tumour control, especially for those with severe cardiovascular involvement and high surgical risk (4).

Current Research Topic remains of high scientific and clinical interest, and includes five papers from well known pituitary research groups from Poland and Brasil.

- The excess GH impact on cardiovascular system is investigated in two papers presenting observational, prospective clinical studies on acromegalic patients vs controls. The paper from Jurek et al. studies the noninvasive cardiovascular assessment using impedance cardiography. The research performed on 33 active acromegaly and 155 controls shows early significant changes of parameters investigated.
- Furthermore, the paper from Gadelha et al. brings into focus the ventricular dysfunction in 25 active acromegaly versus 44 controls, using the modern speckle-tracking echocardiography.
- The other two papers refer to muscular and haematologic impact of acromegaly. Mizera et al. investigates the myokines irisin and myostatin. Authors conclude in a single-center cross-sectional study on 43 acromegalic and 60 controls, that irisin is suppressed in acromegaly independent of the control of the disease, but has no significant impact upon myostatin levels.
- The other paper, by Krygier et al., assesses the hepcidin concentration as well as hematologic parameters in a prospective observational clinical study on 25 active acromegaly vs 25 controls. Authors demonstrate that there is a decreased hepcidin level in acromegalic patients in comparison to the control group.
- The last, fifth paper is a retrospective study carried out by two Polish centers and concerns the use of pasireotide LAR in first-generation SRLs refractory patients. The main conclusion is that pasireotide LAR is an effective therapeutic option in patients with acromegaly refractory to 1^st^ generation SRLs. However, such a therapy may result in pasireotide-associated hyperglycaemia in some patients, which requires an early, aggressive medical therapy to prevent glucose homeostasis alterations.

In the broad portfolio of acromegaly complications, the papers in this group are shedding light on new aspects of acromegaly, aiming an earlier diagnosis, a better control of co-morbidities and treatment strategies.

## Author Contributions

Authors are associate editors of the Pituitary Endocrinology section and edited several papers grouped under the Acromegaly Complications Research Topic. Both authors contributed equally to the editorial.

## Conflict of Interest

The authors declare that the research was conducted in the absence of any commercial or financial relationships that could be construed as a potential conflict of interest.

## Publisher’s Note

All claims expressed in this article are solely those of the authors and do not necessarily represent those of their affiliated organizations, or those of the publisher, the editors and the reviewers. Any product that may be evaluated in this article, or claim that may be made by its manufacturer, is not guaranteed or endorsed by the publisher.
